# Application of Double-Strand RNAs Targeting Chitin Synthase, Glucan Synthase, and Protein Kinase Reduces *Fusarium graminearum* Spreading in Wheat

**DOI:** 10.3389/fmicb.2021.660976

**Published:** 2021-07-09

**Authors:** Peng Yang, Shu-Yuan Yi, Jun-Na Nian, Qing-Song Yuan, Wei-Jie He, Jing-Bo Zhang, Yu-Cai Liao

**Affiliations:** ^1^Molecular Biotechnology Laboratory of Triticeae Crops, Huazhong Agricultural University, Wuhan, China; ^2^College of Plant Science and Technology, Huazhong Agricultural University, Wuhan, China; ^3^Forestry and Fruit Tree Research Institute, Wuhan Academy of Agricultural Sciences, Wuhan, China; ^4^Resource Institute for Chinese & Ethnic Materia Medica, Guizhou University of Traditional Chinese Medicine, Guiyang, China

**Keywords:** *Fusarium graminearum*, RNAi, chitin synthase, glucan synthase, protein kinase, spray-induced gene silencing, wheat

## Abstract

Controlling the devastating fungal pathogen *Fusarium graminearum* (Fg) is a challenge due to inadequate resistance in nature. Here, we report on the identification of RNAi molecules and their applications for controlling Fg in wheat through silencing chitin synthase 7 (Chs7), glucan synthase (Gls) and protein kinase C (Pkc). From transgenic Fg strains four RNAi constructs from Chs7 (Chs7RNAi−1, −2, −3, and −4), three RNAi constructs from Gls (GlsRNAi−2, −3, and −6), and one RNAi construct from Pkc (PkcRNAi−5) were identified that displayed effective silencing effects on mycelium growth in medium and pathogenicity in wheat spikes. Transcript levels of Chs7, Gls and Pkc were markedly reduced in those strains. Double-strand RNAs (dsRNAs) of three selected RNAi constructs (Chs7RNAi-4, GlsRNAi-6 and PkcRNA-5) strongly inhibited mycelium growth *in vitro*. Spray of those dsRNAs on detached wheat leaves significantly reduced lesion sizes; the independent dsRNAs showed comparable effects on lesions with combination of two or three dsRNAs. Expression of three targets Chs7, Gls, and Pkc was substantially down-regulated in Fg-infected wheat leaves. Further application of dsRNAs on wheat spikes in greenhouse significantly reduced infected spikelets. The identified RNAi constructs may be directly used for spray-induced gene silencing and stable expression in plants to control *Fusarium* pathogens in agriculture.

## Introduction

*Fusarium graminearum* (Fg) is a predominant fungal pathogen that causes a devastating Fusarium head blight (FHB) in wheat and other small grain cereals worldwide (Bai and Shaner, 2004). Fg can infect plants from germination to grain filling stages and produce various types of mycotoxins such as deoxynivalenol, nivalenol, and zearalenone during infection (Li et al., 2010; Kazan et al., 2012; Qiu et al., 2019). Cereal grains harvested from Fg-infected wheat in fields are often highly contaminated with Fusarium mycotoxins, which may enter food/feed chains and thus pose a serious health threat to humans and farm animals (Pestka and Smolinski, 2005; Zhang et al., 2013; Jiang et al., 2017; Qiu et al., 2019). FHB epidemics occur frequently in the middle and lower reaches of Yangtze river in China and have now spread to northern China, reaching historically high epidemic acreages in the last few years (Chen et al., 2017; Cheng et al., 2017). FHB has re-emerged as a serious disease in Europe and Northern America since the middle of 1990’s, causing huge yield losses (Windels, 2000; Starkey et al., 2007; Xu and Nicholson, 2009). Cultivation of resistant cultivars is considered the most efficient and environment-friendly strategy to protect crops against pests. However, resistance against FHB in plants is inadequate and currently crop cultivars grown in agriculture have low levels of resistance (Liu, 2001; Bai and Shaner, 2004; Xue et al., 2009). Fungicides have been widely used in fields for many years to control this disease, which can stimulate mycotoxin biosynthesis and produce undesirable environmental consequences and fungicide-resistance Fg strains (Simpson et al., 2001; Chen et al., 2007). Therefore, there is a pressing need to test novel resistance to control Fg-caused diseases in plants.

RNA interference (RNAi) is a powerful tool to silence a gene of interest (Fire, 2007; Melnyk et al., 2011; Fischer, 2015). Expression *in planta* of hairpin RNAi constructs reduces the transcripts of target genes and hence alters plant phenotypes (Tang et al., 2003; Baulcombe, 2004; Baum et al., 2007; Thakare et al., 2017). Host-induced gene silencing (HIGS) of fungal genes has been demonstrated to enhance resistance to the fungal pathogens in plants (Nowara et al., 2010). HIGS of cytochrome P450 lanosterol C14α-demethylase-encoding genes and a chitin synthase 3b gene conferred high resistance against FHB pathogens in barley (Koch et al., 2013) and wheat (Cheng et al., 2015). Spray-induced gene silencing (SIGS) of cytochrome P450 lanosterol C14α-demethylase-encoding genes reduced fungal spreading in barley (Koch and Kogel, 2014; Koch et al., 2016, 2019). RNAi molecules derived from key genes in fungal genome appear to become a rich, useful resistance resource for Fusarium pathogens in plants (Zhang and Ruvkun, 2012; Koch et al., 2013; Ghag et al., 2014; Cheng et al., 2015; Chen et al., 2016; Machado et al., 2018). However, RNAi constructs from different fungal genes varied greatly in their effectiveness for controlling fungal diseases. Nowara et al. (2010) reported that only 16 of 76 putative targets from fungal pathogen *Blumeria graminis* for HIGS in barley had significant impacts on the conidial development in barley. Another chitin synthase member in Fg, Chs5, did not have HIGS effect in wheat (Chen et al., 2016) although it is involved in pathogenicity and development (Kim et al., 2009). Furthermore, different segments from the same genes may have varied and even opposite effects on their targets and phenotypes. Cheng et al. (2015) reported that Chs3bRNAi constructs−1, −2, −3, and −5 of a chitin synthase 3b (*Chs3b*) gene from Fg substantially interfered mycelium growth, and reduced virulence and chitin contents whereas Chs3bRNAi-4 had a similar mycelium growth pattern and significantly increased virulence and chitin contents compared with that of its wild type Fg strain. Therefore, it is essential to select proper RNAi targets and to screen for the most effective RNAi molecules from the targets for SIGS or HIGS to effectively control fungal pathogens in plants.

Chitin and glucan are the key cell wall components of filamentous fungi (Latgé, 2007). They are cross-linked to constitute fungal skeletons to strengthen the cell walls and play key important roles in morphogenesis, adaptation, protection and survival (Latgé, 2007; Aimanianda et al., 2017). Chitin synthase (Chs) and glucan synthase (Gls) are the enzymes responsible for the biosynthesis of chitin and glucan, respectively; these enzymes are essential for fungal development and physiology (Latgé, 2007; Xu et al., 2010; Liu et al., 2016, 2017; Aimanianda et al., 2017; Zhang et al., 2019). Chitin and chitin synthase are absent in plants and animals. Fg genome contains eight *Chs* genes and *Chs7* plays a key role in fungal development and pathogenicity (Kim et al., 2009; Xu et al., 2010; Cheng et al., 2015; Liu et al., 2016). Protein kinase C (*Pkc*) is involved in mitogen-activated protein kinase pathway and plays an important role for the control of cell cycle, distribution of chitin synthases and regulation of oligosaccharyl transferase (Penn et al., 2015; Mishra et al., 2017; Heinisch and Rodicio, 2018). Fg genome contains only one Gls gene and one Pkc gene, and deletion of either Gls or Pkc genes in fungal pathogens generated no viable colonies indicating that they are essential for Fg survival (Wang et al., 2011; Basha et al., 2013). All these three enzymes, Chs, Gls and Pkc, are promising targets for the development of antifungal agents (Basha et al., 2013; Chaudhary et al., 2013; Lin et al., 2017; Wiederhold, 2018). Therefore, interference of biosynthesis of these enzymes may inhibit fungal growth and development. Thus, they may also be used as ideal targets for RNAi interference.

In this study, three Fg genes, *Chs7*, *Gls*, and *Pkc*, were chosen as the targets for RNAi. Each gene sequence was divided into different segments with ∼500 bp to generate RNAi constructs that were genetically transferred into Fg strain 5035. Transgenic Fg strains containing RNAi constructs were comparatively assayed for their mycelium growth, transcript levels and pathogenicity. One most effective RNAi construct from each gene was selected to generate double-strand RNAs that displayed effective silencing effects on Fg spreading in wheat leaves and spikes. The identified RNAi constructs may be directly used as novel resistance germplasms for SIGS and HIGS to control FHB diseases in plants.

## Materials and Methods

### Fungi, Plants, and Culture and Growth Conditions

*Fusarium graminearum* (Fg) strain 5035 was isolated from a scabby wheat spike collected from a field in Wuhan, China (Qu et al., 2008) and used throughout this study. This strain produces DON and 15-ADON. Wild type (WT) strain 5035 and its transgenic strains transformed with RNAi constructs were cultured on potato dextrose agar (PDA). Macroconidia were produced on synthetic nutrient SNA-medium and collected as previously described (Koch et al., 2013). Bread wheat cultivars *Triticum aestivum* L. Annong8455, Fielder and Xiangmai 76 were grown in a greenhouse (22°C, 16 h of light and 8 h of darkness). The wheat cultivar Annong8455 was used for pathogenicity assays of transgenic Fg strains, and the Fielder for spray inoculation and Xiangmai 76 for single floret inoculation with Fg and dsRNAs.

### DNA and RNA Isolation, and Southern Blot

Fungal DNA was extracted from Fusarium mycelia as described previously (Nicholson et al., 1997). Total RNA was extracted from mycelia on PDA and wheat leaves that were sprayed with dsRNA and inoculated with Fg by using TRIzol reagent (Invitrogen, Carlsbad, CA, United States) according to the manufacturer’s instructions. The isolated RNA was transcribed into cDNA as described previously (Cheng et al., 2015) with Revert Aid First strand cDNA thesis kit (Thermo Fisher Scientific, Waltham, United States). The coding sequences of Chs7, Gls, and Pkc were amplified from cDNA transcribed from Fg strain 5035 with primer pairs Chs7cDNAP1/Chs7cDNAP2, GlscDANP1/GlscDNAP2, and PkccDNAP1/PkccDNAP2 (Supplementary Table 1), respectively, and cloned into pMD 18-T Vector (TaKaRa, Dalian, China). PCRs were performed in a PTC-100 Thermal Cycler (MJ Research, Waltham, MA, United States) with gene-specific PCR primers (Supplementary Table 1). Southern blot analyses of 6 selected transgenic Fg strains containing relevant RNAi constructs (2 from each of Chs7RNAi constructs, GlsRNAi constructs and PkcRNAi constructs) were performed with non-radioactive DIG labeling kit (Roche, Mannheim, Germany) as previously described (Song et al., 2014).

### RNAi Constructs

A 3216-bp coding sequence of Chs7 cDNA (accession no. FGSG_12039) amplified from Fg 5035 with primers (Supplementary Table 1) was divided into seven fragments (designated as Chs7-1: 1-582 nt; Chs7-2: 596-1114 nt; Chs7-3: 1072-1606 nt; Chs7-4: 1568-2076 nt; Chs7-5: 2046-2582 nt; Chs7-6: 2546-2961 nt; Chs7-7: 2873-3216 nt; Supplementary Figures 1, 2) to construct seven fungal RNAi constructs (Chs7RNAi-1 to Chs7RNAi-7) using a pSXS-Neo-Tet vector and methods previously described (Cheng et al., 2015). Each fragment was constructed in the vector in sense-intron-antisense manner so that a hairpin structure can be generated after transcription. A cutinase intron sequence from rice blast fungus *Magnaporthe oryzae* (Nakayashiki et al., 2015) was located between sense and antisense fragments (Supplementary Figure 3). The resulting RNAi vectors were used for specific integration into the PLS locus in Fg for functional analysis. This PLS1 gene (FGSG_08695) encoding a tetraspanin (Tet) is a single copy locus and dispensable for growth, reproduction, and plant infection in Fg (Rittenour and Harris, 2008). Similarly, a 6082-bp coding sequence of Gls cDNA (accession no. FGSG_07946) from Fg 5035 was divided into 13 fragments (designated as Gls-1: 216-808 nt; Gls-2: 761-1369 nt; Gls-3: 1309-1831 nt; Gls-4: 1790-2330 nt; Gls-5: 2190-2769 nt; Gls-6: 2654-3216 nt; Gls-7: 3168-3698 nt; Gls-8: 3648-4175 nt; Gls-9: 4115-4766 nt; Gls-10: 4619-5163 nt; Gls-11: 5068-5616 nt; Gls-12: 5513-5992 nt; Gls-13: 5896-6297 nt; Supplementary Figures 1, 2) to construct 13 fungal RNAi constructs (GlsRNAi-1 to GlsRNAi-13) using the same pSXS-Neo-Tet vector. Also a 3504-bp fragment of Pkc cDNA (accession no. FGSG_90606) from Fg 5035 was divided into eight fragments (designated as Pkc-1: 1-299 nt; Pkc-2: 290-835 nt; Pkc-3: 790-1260 nt; Pkc-4: 1221-1624 nt; Pkc-5: 1744-2147 nt; Pkc-6: 2093-2629 nt; Pkc-7: 2590-2990 nt; Pkc-8: 3015-3504 nt; Supplementary Figures 1, 2) to construct eight fungal RNAi constructs (PkcRNAi-1 to PkcRNAi-8) as described above for Chs7RNAi constructs.

### Fungus Transformation, Mycelium Growth, and Pathogenicity

Transformation of Fg strain 5035 and identification of transformants were carried out as described previously (Maier et al., 2005). Transgenic strains were cultured on PDA in the presence of G418 (30 μg/mL) (Roche Diagnostics, Mannheim, Germany) as described previously (Xu et al., 2010) and their mycelium growth was compared with that of WT Fg strain 5035 that grew on PDA in incubator at 22°C for 4 days. Macroconidia of transgenic Fg strains and strain 5035 were cultured in plates containing synthetic nutrient SNA medium (Koch et al., 2013). The pathogenicity assay of transgenic Fg strains and strain 5035 was carried out with wheat cultivars Annong8455 as previously described (Cheng et al., 2015). Twenty spikes (one spike per plant) at flowering stages were inoculated with 10 μl droplet of macroconidia suspension (5 × 10^5^ spores/mL). The inoculated spikes were covered with sealed bags for 3 days. Visually infected spikelets were scored at 21 days after inoculation (dai). The percentages of infected spikelets were calculated.

### *In vitro* Transcription

*In vitro* transcription of fungal RNAi fragments was performed with a T7 RiboMAX^TM^ Express RNAi System (Promega, Madison, United States). DNA templates were generated by flanking the T7 promoter using PCR with the T7 primer (TAATACGACTCACTATAGGGG) appended to specific primers. The *in vitro* transcription reactions were assembled by mixing 1 μg template DNA with 2 μl of ATP, CTP, GTP and UTP (75 mM each) and adding 10 μl of the reaction buffer and 2 μl of the T7 enzyme mixture. The mixture was incubated at 37°C for 3 h; the products were incubated at 75°C for 5 min and then cooled down to room temperature. For removing the DNA template and single-strand RNA, the transcription products were incubated for 30 min after addition of 2 μl of Dnase (2 U/μl) and 1 μl of a 1:200 Rnase A dilution (50 μg/mL). The dsRNAs were purified by isopropanol precipitation and checked on a 1% agarose gel, then quantified using DS11-Spectrophotometer (Denovix, United States).

### Inhibitory Assay

Macroconidia of Fg strain 5035 were collected from plates containing synthetic nutrient SNA medium and diluted to 2 × 10^3^ spores/mL liquid SNA medium (Koch et al., 2013). DsRNAs were diluted to 50 and 100 nM, respectively. 45 μl SNA medium containing diluted macroconidia and 50 μl diluted dsRNAs were added in a 96-well microtiter plate, followed by addition of 5 μl annealing buffer. Equal volumes of Gfp-dsRNA and SNA omitting dsRNAs was used as controls. Each sample was in triplicate. The samples were incubated at 25°C for 16 h and observed with an inverted microscope.

### Spray Inoculation on Wheat Leaves

Detached leaf assay was used for spray inoculation as previously described (Koch et al., 2019) except that wheat leaves were used instead of barley leaves. Wheat leaves were detached from 4-week-old seedlings of wheat cv. Fielder that grew in greenhouse. Detached leaves were placed in square Petri dishes (100 × 100 × 17 mm) containing 1% agar. Each dish contained five leaves whose upper and lower parts were covered with glasses leaving only the middle part (about 1 cm) uncovered. Leaves were sprayed with 500 μl dsRNAs per plate at final concentrations of 50 nM by using a 5 mL-spray flask. Dishes were closed after leaf surface was dried. After incubation in growth chamber at 20°C for 24 h, leaves were inoculated with 20 μl of Fg 5035 macroconidia (2 × 10^4^ spores/mL) and incubated in growth chamber at 20°C. At the fourth day after fungal inoculation (dai), infection symptoms on wheat leaves were scored by measuring lesion sizes with ImageJ software. 20 leaves were inoculated with each dsRNA or dsRNA combination, the percentage of infect area relative to total area of each leaf was calculated, and the ratio were compared with that of controls treated with TE and Gfp-dsRNAs. The experiment was repeated once.

### Single Floret Inoculation on Wheat Spikes

Aliquots of 8 μl dsRNAs at the final concentration of 50 nM in 0.02% tween 20 were injected into the middle florets of wheat spikes (one injection per spike; cv. Xiangmai 76) at flowering stages in greenhouse. RNA injection experiments comprised of single dsRNA (Chs7-4, Gls-6, and Pkc-5) and the combination of two (Chs7-4/Gls-6, Chs7-4/Pkc-5, and Gls-6/Pkc-5) and three dsRNAs (Chs7-4/Gls-6/Pkc-5). Control spikelets were injected with 8 μl Gfp-dsRNAs 0.02% tween 20. Ten spikes were injected for each sample and the experiment was repeated once. The injected wheat spikes were covered with sealed bags to keep humidity for 24 h in greenhouse. Then the florets with dsRNAs were inoculated with 10 μl droplet of Fg 5035 macroconidia (5 × 10^5^ spores/mL). The inoculated wheat plants were kept humid with bags for 3 days. The diseased spikelets were evaluated 14 days post inoculation (dpi) and the percentages of infected spikelets were calculated.

### Mycotoxin Profiling

Mycotoxin contents from the grains of controls and dsRNA treated plants inoculated with Fg strain 5035 were profiled using gas chromatography-mass spectrometry (GC-MS) as previously described (Baldwin et al., 2018).

### Quantitative RT-PCR

For quantitative real-time PCR (qRT-PCR) analysis, macroconidia of transgenic Fg strains and wild type strain 5035 were cultured for 36 h in PDA and the mycedia were collected. Total RNA was extracted using TRIzol reagent. The RNA was subjected to reverse transcription with Superscript III and an oligo-dT 20 random primer according to the instruction of the producer (Invitrogen, Carlsbad, CA, United States). Amplifications were carried out with thermal cycling consisting of 30 cycles of denaturation (94°C, 1.5 min), annealing (55°C, 1 min), and extension (72°C, 2 min). For isolation of total RNA from dsRNA-sprayed wheat leaves, leaves inoculated with Fg strain 5035 for 4 days were ground into powders in liquid nitrogen. RNA extraction, cDNA reverse transcription and qRT-PCR were performed as previously described above (Cheng et al., 2015). Specific primers for the amplification with specific primers (Supplementary Table 1).

### Statistical Analysis

All the data were analyzed using SAS release 6.12 (SAS Institute, Cary, NC, United States) at significance levels of 0.05, 0.01, or 0.001.

## Results

### Generation of Transgenic Fg Strains Containing RNAi Constructs

**FIGURE 1:**
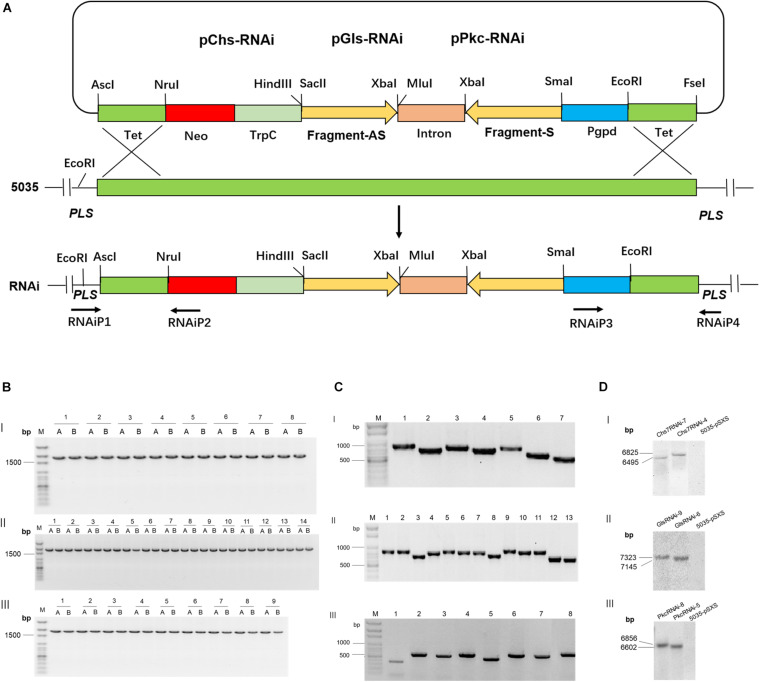
Integration of Chs7RNAi, GlsRNAi and PkcRNAi constructs into *Fusarium graminearum* genome and PCR and Southern blot analyses of transformants. **(A)** A schematic diagram for homologous recombination between the replacement vectors pRNAi series (Chs7RNAi, GlsRNAi and PkcRNAi) and the PLS gene locus of *F. graminearum* strain 5035-pSXS-Neo-Tet. **(B)** PCR analyses of transgenic *F. graminearum* strains carrying Chs7RNAi, GlsRNAi, PkcRNAi and 5035-pSXS-Neo-Tet constructs. Four strains from each transformation were randomly selected for analysis (here only one strain from each transformation is shown as an example). PCR products from the 5′ and 3′ regions of the disrupted PLS gene were amplified with the primers RNAiP1/RNAiP2 and RNAiP3/RNAiP4, respectively. The 1752− and 1777-bp fragments for Chs7 and pSXSH-Neo-Tet (B-I, lane1-7 and lane 8), Gls and pSXSH-Neo-Tet (B-II, lane1-13 and lane 14) and Pkc and pSXSH-Neo-Tet (B-III lane1-8 and lane 9) were amplified from the 5′ and 3′ regions of the disrupted PLS gene. M: 100 bp DNA Marker; A: PCR products from the 5′ regions of the disrupted PLS gene; B: PCR products from the 3′ regions of the disrupted gene. **(C)** Construct sequence-specific PCR. To amplify seven Chs7RNAi construct sequences, seven pairs of primers Chs7SF1/intronP2, Chs7SF2/intronP2, Chs7SF3/intronP2, Chs7SF4/intronP2, Chs7SF5/intronP2, Chs7SF6/intronP2, and Chs7SF7/intronP2 were used, generating seven products of 759 bp (Chs7-1), 695 bp (Chs7-2), 711 bp (Chs7-3), 685 bp (Chs7-4), 713 bp (Chs7-5), 592 bp (Chs7-6), and 520 bp (Chs7-7), respectively. For PCR of Gls construct sequences, 13 pairs of primers GLSF1/intronP2, GLSF2/intronP2, GLSF3/intronP2, GLSF4/intronP2, GLSF5/intronP2, GLSF6/intronP2, GLSF7/intronP2, GLSF8/intronP2, GLSF9/intronP2, GLSF10/intronP2, GLSF11/intronP2, GLSF12/intronP2, GLSF13/intronP2 were used to generate 13 PCR products of 759 bp (Gls-1), 785 bp(Gls-2), 699 bp (Gls-3), 718 bp (Gls-4), 756 bp (Gls-5), 739 bp (Gls-6), 707 bp (Gls-7), 704 bp (Gls-8), 828 bp (Gls-9), 721 bp (Gls-10), 725 bp (Gls-11), 656 bp (Gls-12), and 578 bp (Gls-13), respectively. Similarly, eight PCR primer pairs for PkcRNAi were used to generate eight specific products of 448 bp (Pkc-1), 698 bp (Pkc-2), 630 bp (Pkc-3), 723 bp (Pkc-4), 562 bp (Pkc-5), 696 bp (Pkc-6), 666 bp (Pkc-7), 689 bp (Pkc-8), respectively. All these PCR products have the sizes as expected. **(D)** Southern blot analyses of selected transgenic *F. graminearum* strains carrying Chs7-, Gls- and PkcRNAi constructs. The genome DNA isolated from strains 5035-pSXS-Neo-tet, Chs7RNAi-4, Chs7RNAi-7, GlsRNAi-6, GlsRNAi-9, PkcRNAi-5 and PkcRNAi-8 were digested with *Eco*RI and hybridized with a fragment of a kanamycin resistance gene amplified with the primers KamP1/KamP2. The sizes of hybridization fragments for the strains Chs7RNAi-4, Chs7RNAi-7, GlsRNAi-6, GlsRNAi-9, PkcRNAi-5 and PkcRNAi-8 were 6825 bp, 6495 bp, 7145 bp, 7323 bp, 6602 bp, and 6856 bp, respectively, as expected.

Three genes that encode a chitin synthase 7 (Chs7, FGSG_12039), a glucan synthase (Gls, FGSG_07946), and a protein kinase C (pkc, FGSG_90606), were selected as RNAi targets. To generate RNAi constructs with ∼500 bp, the coding sequences of Chs7 (from 1 to 3216 nt), Gls (from 216 to 6297 nt), and Pkc (1 to 3504 nt) were divided into seven fragments (Chs7-1 to Chs7-7), 13 fragments (Gls-1 to −13), and eight fragments (Pkc-1 to −8), respectively (Supplementary Figures 1, 2). These fragments were amplified by PCRs with the specific primers (Supplementary Table 1) with cDNA transcribed from mRNA isolated from Fg strain 5035 as templates. Each of the amplified PCR fragments was constructed in an RNAi expression vector pSXS in Fg with a sense and an anti-sense orientation between which there was intron sequence (Supplementary Figure 3); this design of RNAi constructs gave rise to a double-strand RNA sequence with a hairpin structure after transcription. Searching for off-target sequences in plants in SI-FI database^1^ revealed that no off-target sequences were found in wheat, barley, maize, and/or other plant species for coding sequences of Chs7, Gls and Pkc (Supplementary Table 2). The generated RNAi constructs (Chs7RNAi-1 to Chs7RNAi-7; GlsRNAi-1 to GlsRNAi-13; PkcRNAi-1 to PkcRNAi-8) were transformed into Fg strain 5035 via homologous recombination and the transgenic Fg strains designated grown in PDA media in the presence of antibiotics were selected. PCR identification indicated that all transgenic Fg strains contained respective RNAi constructs integrated into a PLS site in Fg genome as expected (Figures 1A-C) and are used for subsequent studies.

### Screening for RNAi Constructs Inhibiting Fg Growth in Medium

**FIGURE 2:**
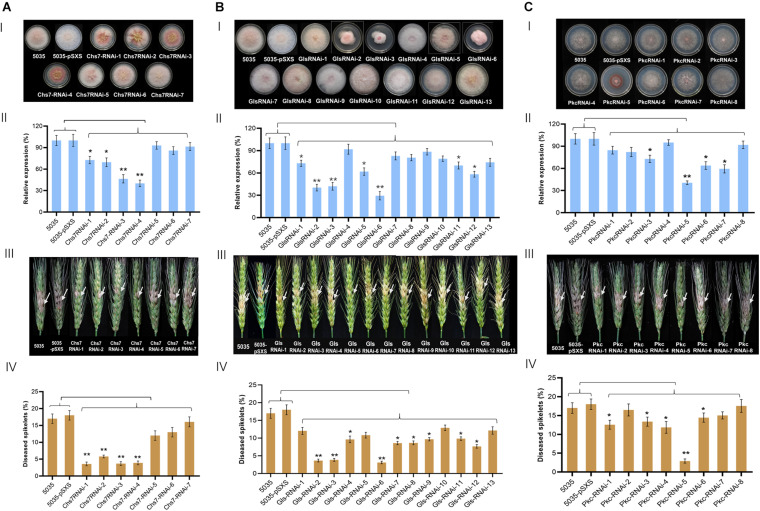
Mycelium growth, relative expression of target genes and pathogenicity of Fg strains transformed with RNAi constructs derived from Chs7 **(A)**, Gls **(B)**, and Pkc **(C)**. **I**, Mycelium growth of *Fusarium graminearum* (Fg) strains on PDA at 22°C for 4 days. **II**, Relative expression levels of *Chs7*, *Pkc*, and *Gls* genes in the Fg strains shown in **I**. The expression levels were normalized to the levels of the fungal β*-tubulin* gene. **III**, Phenotypes of wheat spikes at 21 dai with Fg strains shown in **I**. **IV**, Percentages of infected wheat spikelets at 21 dai. The bars represent mean values standard error of three independent biological experiments **II** and 20 wheat spikes **III**. Asterisks represent a statistically significant difference (*P < 0.05 and **P < 0.01), according to Student’s *t*-tests.

To identify which of RNAi constructs were most effective in silencing target genes, we carried out comparative analyses of mycelium growth of all RNAi transgenic Fg strains from each gene with their wild type strain 5035 on media. A very substantial variation of mycelium growth and colony size was observed between different RNAi constructs from the same gene as well as between RNAi constructs from different genes. Of seven Chs7RNAi construct-transgenic Fg strains that were cultured on PDA media for 3 days, Chs7RNAi−1, −2, −3, and −4 displayed abnormal mycelium colonies, with smaller colony sizes and much less aerial mycelia than WT strain 5035 and 5035 containing vector pSXS-Neo-tet, whereas Chs7RNAi−5, −6, and −7 strains had comparable mycelial growth as their WT 5035 and 5035 with the empty vector. (Figure 2A-I). As for 13 GlsRNAi constructs, GlsRNAi−2, −3, and −6 had markedly abnormal colonies, with small sizes and short, dense aerial mycelia (Figure 2B-I); the remaining ten transgenic strains had comparable colony sizes although there are some morphological variations. For eight PkcRNAi constructs, only PkcRNAi-5 had very much different colony size and morphology compared with WT 5035 strain and 5035 with vector pSXS-Neo-tet (Figure 2C-I), while other seven RNAi transgenic strains exhibited slightly variations and/or similar morphological features.

### Transcript Levels of Target Genes in Transgenic Fg Strains

To determine whether the expression of the target genes in transgenic Fg strains was changed, qRT-PCR was used to measure the transcript levels of the target genes in the transgenic and non-transgenic WT strains. WT strain and WT strain contain empty vector pSXS-neo-tet expressed target transcripts at comparable levels. Relative to these two strains, two Chs7RNAi-transgenic strains (Chs7RNAi−3 and −4) had very significant reduction of Chs7 transcript levels, ranging from 54% in Chs7RNAi-1 (i.e., 46% of the 5035 Chs7 level) to 60% in Chs7RNAi-4 (Figure 2A-II), and other two strains, Chs7RNAi−1 and −2, had significant transcript reduction. A very significant reduction of Gls transcripts was observed in three GlsRNAi-transgenic strains (GlsRNAi−2, 60%; GlsRNAi−3, 58%; GlsRNAi−6, 71%) (Figure 2B-II). As for the transcript levels of Pkc, one transgenic strain PkcRNAi-5 displayed very significant 59% reduction and other three strains (PkcRNAi−3, −6, and −7) had significant reduction of 27% (PkcRNAi−3), 36% (PkcRNAi-6), and 41% (PkcRNAi-7), respectively (Figure 2C-II). These results indicated that different RNAi constructs derived from the same gene had markedly different efficacies for down-regulation of their respective targets, and among them, Chs7RNAi−3 and Chs7RNAi−4, GlsRNAi−2, GlsRNAi−3 and GlsRNAi−6, and PkcRNA−5 had more efficient efficacy than other countpartner constructs assayed.

To further confirm that a single integration took place in the transgenic Fg strains, Southern blots were performed using DNA isolated from six representative transgenic Fg strains (Chs7RNAi−4 and −7; GlsRNAi−6 and −9; and PkcRNAi−5 and −8) and WT strain 5035. The results showed that all these transgenic strains contained only one band with expected sizes (Figure 1D), demonstrating the presence of a single copy of transgenes in the PLS site in Fg genome.

### Pathogenicity of RNAi-Transgenic Fg Strains on Wheat Spikes

To assess the impact of RNAi constructs on pathogenicity, macroconidia spores of all the transgenic Fg strains, WT strain 5035 and 5035 strain contain empty vector pSXS-neo-tet were used to inoculate wheat spikes, and the percentages of infected spikelets were evaluated at 21 days after inoculation (dai). Among seven Chs7RNAi-transgenic Fg strains, four strains, Chs7RNAi−1, −2, −3, and −4, had 5% to 7% infected spikelets (Figures 2A-III,IV), with a very significant reduction of 67%-79% compared with WT strain 5035 (17%) and 5035 with pSXS-Neo-Tet. The other three transgenic strains Chs7RNAi−5, −6, and −7 showed a high level of infected spikelets similar to that of WT strain. From 13 GlsRNAi-transgenic strains, three of them, GlsRNA−2, −3 and −6 had 3.1% to 3.9% infected spikelets, with a very significant reduction of 72%-78% compared with that of WT strain 5035 (14.1%); six other strains had significant reduction of infected spikelets (GlsRNAi−4, 31%; GlsRNAi−7, 39%; GlsRNAi−8, 38%; GlsRNAi−9, 31%; GlsRNAi−11, 30%; GlsRNAi−12, 45%). As for PkcRNAi-transgenic strains, one strain PkcRNAi−5 had the lowest infected spikelets (2.9%), with a very significant 86% reduction relative to that of WT 5035 strain (21.4%); four strains showed significant 32%−44% reduction of infected spikelets (PkcRNAi−1, 41%; PkcRNAi−3, 37%; PkcRNAi−4, 44%; PkcRNAi−6, 32%). Thus, different RNAi constructs expressed in Fg strains showed substantially varied impacts on pathogenicity in wheat spikes; at least one RNAi construct derived from each of three targets Chs7, Gls and Pkc, had very significant impacts on pathogenicity in wheat.

### Inhibitory Effects of *in vitro* Transcribed Double-Strand RNAs on Fungal Growth

**FIGURE 3:**
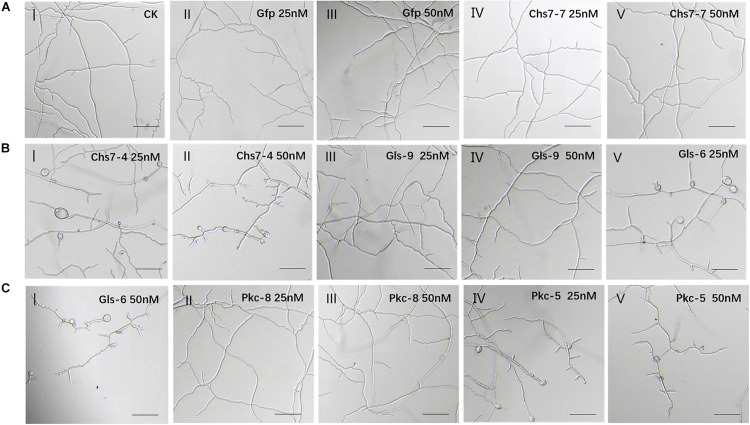
**(A-C)** Inhibition and morphology of *Fusarium graminearum* (Fg) cultured in SNA medium containing dsRNAs. Mycelia at 16 h of incubation with different concentrations of dsRNAs were made. One hundred Fg macroconidia were suspended in 100 μl of liquid SNA medium containing different concentrations of dsRNAs. SNA omitting dsRNAs and Gfp-dsRNAs was used as a negative control.

To reveal visual inhibitory effects of dsRNAs on Fg, double-strand RNAs (dsRNA) were generated by *in vitro* transcription and used for inhibitory assays. Six RNAi constructs, three with effective silencing efficacy (Chs7-4, Gls-6, and Pkc-5) and three with no significant silencing effects (Chs7-7, Gls-9, and Pkc-8) illustrated in Figure 2, were selected. In addition a non-homologus Gfp-dsRNA was used as a negative control. The sequences of these seven RNAi constructs were amplified by PCR and used as templates for *in vitro* transcription with two primers carrying T7 promoter sequences generating dsRNAs (Supplementary Table 1). The generated dsRNAs were individually incubated with macroconidia spores of WT Fg strain 5035 and visualized under microscopy. After incubation for 16 h, macroconidia in controls with Gfp-dsRNAs at a concentration of 25 and 50 nM germinated and mycelia grow quickly with smooth shape and stretches with various branches being spreading, and this growth habit is similar to that of macroconidia with no dsRNA (Figure 3A-I-III). However, when macroconidia were incubated with dsRNAs of either Chs7-4, or Gls-6, or Pkc-5 at a concentration of 25 nM, severe distortion of mycelia was observed in all treatments; mycelia are short and crooked, with various branches, bubbles and uneven surfaces(Figures 3B-I,V,C-IV). At a concentration of 50 nM, these inhibitor effects were even more severe, with further short mycelia and more bubbles on mycelia(Figures 3B-II,C-I,V). On the other hand, all the treatments with dsRNAs of Chs7-7, Gls-9 and Pkc-8 at both concentrations showed similar phenotypes as controls did. These results indicated that only dsRNAs of Chs7-4, Gls-6 or Pkc-5 efficiently inhibited Fg mycelium growth and these three dsRNAs were used for subsequent studies.

### Spray-Induced Gene Silencing of Target Genes on Wheat Leaves

**FIGURE 4:**
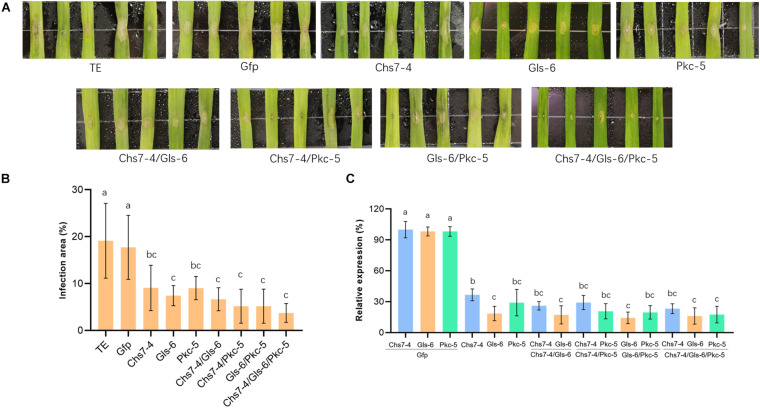
Infection of *Fusarium graminearum* (Fg) on wheat leaves sprayed with different dsRNAs. **(A)** Phenotypes of representative wheat leaves sprayed with dsRNAs and inoculated with Fg at 3 dai. **(B)** Infection area, illustrated as percentage of the total area from 20 leaves for each dsRNA and TE control at 3 dai. Error bars indicate standard errors (SE) of two independent experiments. Different letters indicate statistical significance (*p* < 0.01; ANOVA). **(C)** Expression of *Chs7*, *Gls*, and *Pkc* genes. Relative gene expression was assayed by qRT-PCR and normalized to fungal β-*tubulin* as a reference gene. Total RNA was extracted from detached wheat leaves sprayed with different dsRNAs and inoculated with Fg at 3 dai and reversely transcribed into cDNA. Error bars indicate SE of two independent experiments. Different letters indicate statistical significance (*p* < 0.01; ANOVA).

To evaluate the efficacy of dsRNAs to inhibit Fg *in planta*, four dsRNAs transcribed from Gfp, Chs7-4, Gls-6, and Pkc-5 were sprayed on detached wheat leaves that were then inoculated with macroconidia spores of Fg strain 5035. Chs7-4, Gls-6, and Pkc-5 were sprayed either individually or at different combinations of two/three dsRNAs. After further incubation for 3 days, lesion areas on wheat leaves were measured and the percentages of the lesion areas relative to the whole leaf areas were calculated. The results showed significant differences of lesion areas between CK and treatments (Figure 4A). Control leaves sprayed with TE buffer and Gfp-dsRNAs had 19.1% and 17.7% infected areas, respectively, whereas 7.43%-9.03% lesion areas were observed in leaves sprayed with dsRNAs from individual Chs7−4, Gls−6 or Pkc−5, with reduction of 48%−58%. Leaves sprayed with combinations of two dsRNAs (Chs7-4/Gls-6, Chs7-4/Pkc-5, or Gls-6/Pkc-5) had lesion areas ranging from 5.18% to 7.43%, with reduction of 62%-70% compared with that of controls. More importantly, spray with a combination of three dsRNAs (Chs7-4, Gls-6, and Pkc-5) resulted in 3.73% lesion areas, with 78% reduction (Figure 4B). These results indicated that dsRNAs can efficiently inhibit Fg spreading in wheat leaves and each combination form of dsRNAs had a stronger effects on Fg in wheat leaves.

Transcript levels of fungal targets Chs7, Gls and Pkc in the inoculated wheat leaves were quantitatively assayed by RT-PCR. The three targets in all the dsRNA-sprayed wheat leaves (one dsRNA, two dsRNA, or three dsRNA) were very significantly down-regulated, ranging from 63% to 85% relative to that of controls sprayed with TE buffer or Gfp-dsRNAs, and each target gene expressed transcripts at comparable levels (Figure 4C). These results showed effective SIGS efficacies of three targets in Fg during its infection of wheat.

### Effects of dsRNAs on Fusarium Head Blight in Wheat

**FIGURE 5:**
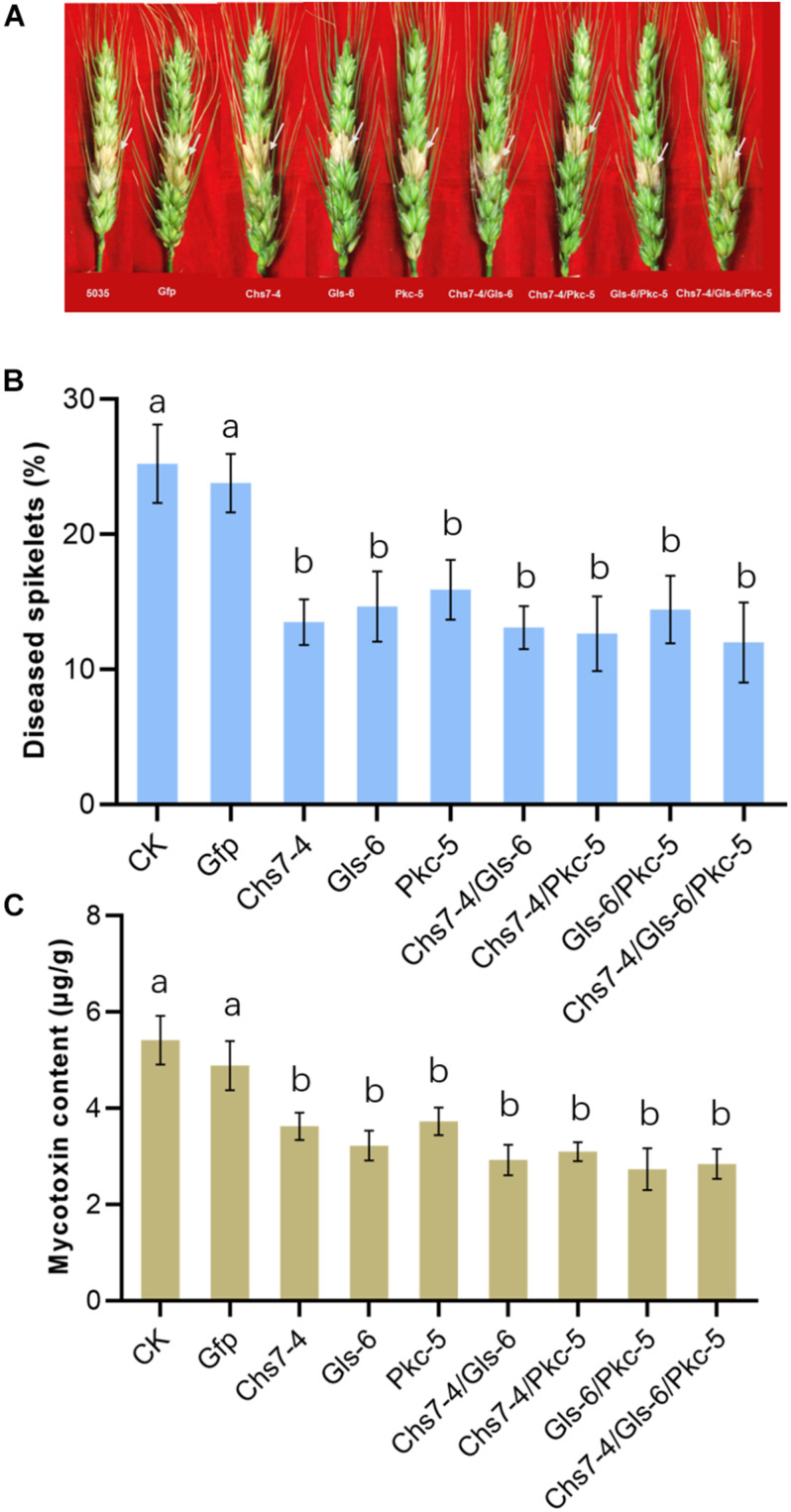
**(A-C)** Disease symptoms, scores and mycotoxin contents of different dsRNA treatments and controls after inoculation with *Fusarium graminearum*. Phenotypes of representative wheat Fusarium head blight at 14 dai are in the top of the panel, the disease scores, expressed as percentages of infected spikelets are in the middle of the panel, and mycotoxin contents of the grains are in the bottom of the panel. Error bars indicate SE of two independent experiments. Different letters indicate statistical significance (*p* < 0.01; ANOVA).

To further reveal effects of dsRNAs on Fusarium head blight in wheat plants, three dsRNAs Chs7-4, Gls-6 and Pkc-5 or their combinations were injected into spikelets of wheat in greenhouse that were then inoculated with macroconidia spores of Fg strain 5035, and mycotoxin amounts of DON in the mature plant grains were determined. Seven different dsRNA treatments (Chs7-4, Gls-6, Pkc-5, Chs7-4/Gls-6, Chs7-4/Pkc-5, Gls-6/Pkc-5, and Chs7-4/Gls-6/Pkc-5) were used, with TE buffer and Gfp-dsRNA were used as negative controls. Percentages of infected spikelets in the dsRNA treatments and controls greatly varied at 14 dpi (Figure 5). Compared to that of controls, single RNA treatments had a significant reduction of infected spikelets from 38% to 46% while the combinations of two or three dsRNAs in one treatment had 43% to 54% reduction. DON contents of the plants treated with dsRNA samples ranging from 2.7 to 3.7 μg/g, significantly lower than that of the two CK plants with 5.4 μg/g and 4.9 μg/g respectively. These results indicated that dsRNAs effectively inhibited Fusarium spreading on wheat spikes and toxin production, and the effect of single dsRNA was comparable with dsRNAs mixed with two or three.

## Discussion

In this study, comparative assays of transgenic Fg strains carrying different RNAi constructs with sense-intron-antisense structures amplified from Fg genome revealed that four RNAi constructs from Chs7, three from Gls and one from Pkc displayed substantial impacts on fungal mycelium growth and pathogenicity. DsRNAs transcribed from three selected RNAi constructs efficiently inhibited Fg growth *in vitro* and in infected wheat leaves and spikes. Expression analyses with transgenic Fg strains and Fg-infected wheat leaves demonstrated a significant reduction of transcript levels for all the three target genes. These results clearly indicated an efficient silencing efficacy for the dsRNAs targeting Chs7, Gls, and Pkc in Fg, suggesting a promising potential for the use of the identified RNAi constructs to control FHB pathogens in agriculture.

Endogenous expression of RNAi constructs in Fg strain via transgenic approach is essential for the identification of the most effective RNAi constructs from a given target. Fungi such as Fusarium species carry all machineries to generate small interfere RNA (siRNA) molecules (Koch et al., 2013; Koch and Kogel, 2014; Weiberg et al., 2014). All RNAi constructs were integrated into the same locus, PLS1, in the genome of Fg strain 5035. A PLS1 gene codes for a tetraspanin that is a single locus and a dispensable in Fg genome; this unique locus often serves as the target site for integration of other genes in relevant Fg strains for functional analyses (Rittenour and Harris, 2008; Song et al., 2014). Different RNAi constructs were integrated into the same PLS1 locus as a single integration as revealed by specific PCR and Southern blot analyses (Figure 1). Thus, phenotypic variations of different RNAi-transgenic Fg strains observed on PDA plates (Figures 2A-I,B-I,C-I) reflected the interfering effects of the integrated RNAi constructs; this is further supported by expression patterns of the target genes showing significant reduction in relevant RNAi-transgenic strains relative to that of WT strain 5035 (Figures 2A-II,B-II,C-II). Fg strains can be easily genetically manipulated due to its high efficiency of stable transformation and simple cultivation in PDA medium. Silencing efficacy of the identified RNAi molecules from transgenic Fg strains were further studied in subsequent assays with *in vitro* inhibition, SIGS in wheat leaves and floret injection in wheat spikes. This screening strategy is very efficient and valuable for the identification of effective RNAi constructs from Fg genome as novel resistance molecules for use to combat FHB pathogens. This may be particularly important for wheat and other small grain cereals where there is a lack of resistance germplasms against FHB. Moreover, it is well known that generation of transgenic wheat or barley is a time-consuming, tedious work, and it is unlikely to evaluate silencing efficacy of different RNAi constructs through transgenic cereal plants. Thus, the current study may represent a good option for rapid screening for effective RNAi molecules from Fg.

Exogenous application of *in vitro* transcribed dsRNAs in wheat leaves and spikes provided laboratory− and greenhouse-based experimental evidences for the use of RNAi molecules to control Fg-caused diseases. Small interfere RNAs (siRNAs, 19-23 nt) present in a culture medium can actively migrate into Fg cells, as visualized by a fluorescence microscope (Cheng et al., 2015). In SNA medium containing dsRNAs of 403-562nt in length (Figure 3 and Supplementary Figure 1), mycelium growth was severely inhibited, especially at concentrations of 50 nM dsRNAs (Figure 3). These results suggested that not merely siRNAs, long dsRNA molecules outside cells can also actively enter into Fusarium cells where fungal RNAi machinery processed dsRNAs into siRNAs that interfered their targets (Koch et al., 2016, 2019). In wheat leaves sprayed with dsRNAs, at the 4th day after inoculating, lesion sizes showed significant reduction compared with that of controls (Figure 4). Annette et al. reported that non-homologous dsRNAs induce PTI responses in plants, including MPK activation, ethylene production, response gene expression and plant growth inhibition (Annette et al., 2016). In this study, compared with TE sprayed leaves, leaves sprayed with non-homologous Gfp-dsRNAs showed comparable levels of growth and fungi infection status, indicating unconspicuous PTI related influence in wheat leaves by non-homologous dsRNAs. Markedly down-regulation of the target genes at this stage of wheat leaves revealed by RT-PCR (Figure 4) appeared to provide molecular support for the view that fungal siRNAs present in plant cells efficiently migrated into fungal cells where they targeted fungal target sequences and thereby interfered their functionality. Koch et al. (2019) reported that SIGS was more efficient than HIGS in down-regulation of target gene expression level. Here down-regulation of target gene expression levels in spray treatments with one species dsRNA ranged from 63% to 85% (Figure 4), which seems to be also higher than HIGS of a *Chs3b* gene (42% to 57%) previously reported (Cheng et al., 2015). This may be a common feature for SIGS, suggesting that SIGS may be further utilized in controlling Fusarium pathogens.

Consistent gene silencing effects in both endogenous expression and exogenous application of RNAi molecules indicated that each of the three genes assayed in this study was a proper target for gene silencing in Fg. Furthermore, spray and injection with a mixture of dsRNAs targeting three different genes gave rise to a corresponding disease and toxin reduction with one, two or three species of dsRNAs (Figures 4A,B, 5), while down-regulation of transcript levels for individual genes was comparable in spray treatments of one, two, or three dsRNAs (Figure 4C). These results suggested that different dsRNA molecules independently targeted respective targets and simultaneously silencing of three genes could generate consistent silencing effect resulting in a smaller lesion size. Koch et al. identified that the growth of Fusarium strain reduced at a cyp51-dsRNA concentration of 37.5 nM, and the mycelia growth was similar with that of Fusarium strain treated with DMI fungicides (Koch et al., 2013). In this experiment, strain spreading can be inhibited on leaves and spikes treated with 50 nM dsRNAs, indicating the SIGS can be applied at this concentration level. Taken together, these features suggest that interfering crucial structural components through RNAi strategy may be an effective way to control fungal pathogens. Therefore, the three different RNAi constructs may be combined for the use in SIGS and HIGS to generate long-last resistance against FHB pathogens in agriculture.

## Data Availability Statement

The raw data supporting the conclusions of this article will be made available by the authors, without undue reservation.

## Author Contributions

Y-CL and J-BZ conceived the experiments. PY, S-YY, and J-NN conducted the experiments. S-YY, J-BZ, and Y-CL analyzed the results. Y-CL, PY, and S-YY wrote the manuscript. W-JH, Q-SY, and J-BZ revised the manuscript. All authors reviewed the manuscript.

## Conflict of Interest

The authors declare that the research was conducted in the absence of any commercial or financial relationships that could be construed as a potential conflict of interest.

## Footnotes

1 http://labtools.ipk-gatersleben.de/
