# Supplementary material for: Application of Double-Strand RNAs Targeting Chitin Synthase, Glucan Synthase, and Protein Kinase Reduces Fusarium graminearum Spreading in Wheat
Source: Front Microbiol. 2021 Jul 9;12:660976. doi: 10.3389/fmicb.2021.660976 (PMC8299488; doi:10.3389/fmicb.2021.660976)
Supplement: Supplementary file 1 [file Data_Sheet_1.doc]

**Supplementary Materials**

**Table S1 Primers used for PCR of fragments derived from genes *Chs7*, *Gls* and *Pkc* to generate for RNAi constructs for transformation of *Fusarium graminearum***

| **Primer** | **Sequence** |
| --- | --- |
| Chs7SF1 | TTAACCCGGGATGGCGAGACGAATGTC |
| Chs7SR1 | GGGTCTAGAGGCGGATGTGAGCAAATAG |
| Chs7SF2 | TAACCCGGGGAGCGAGAGTTTCTAGGC |
| Chs7SR2 | GGATCTAGAAGAGCGGGTTCGGGGACT |
| Chs7SF3 | TTACCCGGGACCGTTTCCATCACTGTT |
| Chs7SR3 | GGGCTCTAGATCTGTTACGACGGTTGGC |
| Chs7SF4 | TACCCGGGCTTGGCGTCTTCCTGAAT |
| Chs7SR4 | GGGTCTAGAGTAGCGGGCTGAGCATTG |
| Chs7SF5 | TAACCCGGGATCTGCTATGCGCAATGC |
| Chs7SR5 | GGCTCTAGATACCAGCGAGGGGCAACA |
| Chs7SF6 | TAACCCGGGAGACCACCAAGACGAACA |
| Chs7SR6 | GCCCTCTAGAACCATGTCCTCGTTCAAG |
| Chs7SF7 | TTCCCCGGGCTTACGGATTATCTGCAC |
| Chs7SR7 | CGGTCTAGAAAGGACGTCGCTTGGAAC |
| Chs7AF1 | CCTAAGCTTTTGCCGCGGATGGCGAGACGAATGTCC |
| Chs7AR1 | GAGGTCGACCAAACGCGTGGCGGATGTGAGCAAATAG |
| Chs7AF2 | TAACCGCGGGAGCGAGAGTTTCTAGGC |
| Chs7AR2 | TAAACGCGTAGAGCGGGTTCGGGGACT |
| Chs7AF3 | TACCCGCGGACCGTTTCCATCACTGTT |
| Chs7AR3 | CCTACGCGTTCTGTTACGACGGTTGGC |
| Chs7AF4 | TTACCGCGGCTTGGCGTCTTCCTGAAT |
| Chs7AR4 | TTAACGCGTGTAGCGGGCTGAGCATTG |
| Chs7AF5 | TAACCGCGGATCTGCTATGCGCAATGC |
| Chs7AR5 | TATACGCGTTACCAGCGAGGGGCAACA |
| Chs7AF6 | CAACCGCGGAGACCACCAAGACGAACA |
| Chs7AR6 | GCGACGCGTACCATGTCCTCGTTCAAG |
| Chs7AF7 | CTCCCGCGGCTTACGGATTATCTGCAC |
| Chs7AR7 | TGAACGCGTAAGGACGTCGCTTGGAAC |
| GLSF1 | TTAACCCGGGCCCCAGCAAATGTCTCGAG |
| GLSR1 | TTACCGCGGTTGTAATCCATCTCGGCAGC |
| GLSF2 | TTAACCCGGGCCAACGTTCAGCGACTTCAC |
| GLSR2 | TTACCGCGGTCGTCACCTTCGAGATC |
| GLSF3 | TTAACCCGGGCCAGAAGAAAGACCCAGAGG |
| GLSR3 | TTACCGCGGAGCACTTCTTCCAGTTCAC |
| GLSF4 | TTAACCCGGGCGTGTGAAGCTCAAGGACGT |
| GLSR4 | TTACCGCGGTATGCTGGCAACATACTTCCTG |
| GLSF5 | TTAACCCGGGGGTGGTGGAAAGAAGATCGA |
| GLSR5 | TTACCGCGGTGGGTTTGTATTTGATCTCC |
| GLSF6 | TTAACCCGGGCGATCGGATCTTCCATCTTGA |
| GLSR6 | TTACCGCGGATCTCATCTGCCAGAATCTTG |
| GLSF7 | TTAACCCGGGGCCTCATGAGTGGGATTG |
| GLSR7 | TTACCGCGGAACCTCAGAGTGACCATC |
| GLSF8 | TTAACCCGGGCAAGAACCTCGTCTGTACTC |
| GLSR8 | TTACCGCGGTTCGTTCAAGTGAAGACC |
| GLSF9 | TTAACCCGGGCATCTTTATGACGACTCGAGG |
| GLSR9 | TTACCGCGGAATGAAAGCACGCCAAATG |
| GLSF10 | TTAACCCGGGTCATTCCCCTTATTGTC |
| GLSR10 | TTACCGCGGGAAGCGTGGCTGCGGGA |
| GLSF11 | TTAACCCGGGCTTCATCTTGGATCTCGT |
| GLSR11 | TTACCGCGGACAGGTCGAAACAATAAGC |
| GLSF12 | TTAACCCGGGGCATGTTCATTCTGGAGAG |
| GLSR12 | TTACCGCGGAAACTACAGCAGGTCCAGCA |
| GLSF13 | TTAACCCGGGGAAACAGTCCAAGCTTAG |
| GLSR13 | TTACCGCGGACTTTCTTCTGTGCATAGG |
| PKCSF1 | CCAACGCGTATGAACGACGATGACG |
| PKCSR1 | TCACCGCGGGATTGCTGGAGG |
| PKCSF2 | TGTCCGCGGGAGATAGAGGTTCC |
| PKCSR2 | GACACGCGTCAACATCTTTGATTGCTA |
| PKCSF3 | ATTCCGCGGCTCAGTGGACAACTCTC |
| PKCSR3 | ATATACGCGTGGGACCAGCAGCTC |
| PKCSF4 | ATACCCGGGCCAAGGGGATGATACT |
| PKCSR4 | GCCCTCTAGATCGGGGACAAGATGA |
| PKCSF5 | TATCCGCGGTCTTACTGCCCACG |
| PKCSR5 | TGTACGCGTATATCGCCCAAAGTCTGA |
| PKCSF6 | AATCCGCGGGCTATGTCTGGAGG |
| PKCSR6 | CGCACGCGTCTCCTTCTTGAGAAC |
| PKCSF7 | ATTCCGCGGCTCTCGAAAGCTCTAT |
| PKCSR7 | GTCTACGCGTATCAGCACACCAAATG |
| PKCSF8 | AATACGCGTTCCCGAATTCATGGC |
| PKCSR8 | ACTCCGCGGTTAGTCAAAATCAGCTGT |
| intronP1 | TTGCTGGAGGATACAGGTGAGC |
| intronP2 | CTGCCGTTCCCTGGCTGTGTGTT |
| KamP1 | AGAAGGCGATGCGCTGCGAAT |
| KamP2 | ATCTGGACGAAGAACATCAGG |
| Chs7-qP1 | ATGGCGAGACGAATGTCC |
| Chs7-qP2 | CGAGCAGCGAGGGTAGCA |
| Gls-qP1 | GACCAGAGTCACCTCTCC |
| Gls-qP2 | GCCTCCCTGAGGGTGGCC |
| Pkc-qP1 | CGCGGGAGATAGAGGTTC |
| Pkc-qP1 | GAACTGGATCTGGGACAA |
| Tublin-F | CGTCCAGAGCAAGAACTCATCA |
| Tublin-R | TGCGTCGGAACATAGCAGTAA |

**Table S2** Prediction of the off-targets of siRNAs belonging to Chs7RNAi4, GlsRNA6 and PkcRNAi5

| **Organism**^†^ | **genome** | Chs7RNAi4 | | GlsRNA6 | | PkcRNAi5 | |
| --- | --- | --- | --- | --- | --- | --- | --- |
|  |  | All hit^‡^ | Efficient  hits^§^ | All hit^‡^ | Efficient  hits^§^ | All hit^‡^ | Efficient  hits^§^ |
| Triticum aestivum | GCA_002220415.3 | 0 | 0 | 0 | 0 | 0 | 0 |
| Arabidopsis thaliana | GCF_000001735.4 | 0 | 0 | 0 | 0 | 0 | 0 |
| Oryza sativa | GCA_014636035.1 | 0 | 0 | 0 | 0 | 0 | 0 |
| Zea mays | GCF_902167145.1 | 0 | 0 | 0 | 0 | 0 | 0 |
| Hordeum vulgare | GCA_004114815.1 | 0 | 0 | 0 | 0 | 0 | 0 |
| Bactrocera dorsalis | GCF_000789215.1 | 0 | 0 | 0 | 0 | 0 | 0 |
| Blumeria graminis | GCA_900519115.1 | 0 | 0 | 0 | 0 | 0 | 0 |

Notes: †*Triticum aestivum, Arabidopsis thaliana, Oryza sativa, Zea mays, Hordeum vulgare, Bactrocera dorsalis* and *Blumeria graminis* gene sequence files were obtained from NCBI Genome Assembly/Annotation Projects (ftp://ftp.ncbi.nlm.nih.gov/genomes/).
‡ Number of siRNAs (21 nt long) with perfect homology to the genome of a respective organism
§ number of siRNAs (21 nt long) with perfect homology to the genome of a respective organism and fulfill some additional criteria of efficient RNAi.
