# Supplementary figures and images for: Application of Double-Strand RNAs Targeting Chitin Synthase, Glucan Synthase, and Protein Kinase Reduces Fusarium graminearum Spreading in Wheat
Source: Front Microbiol. 2021 Jul 9;12:660976. doi: 10.3389/fmicb.2021.660976 (PMC8299488; doi:10.3389/fmicb.2021.660976)

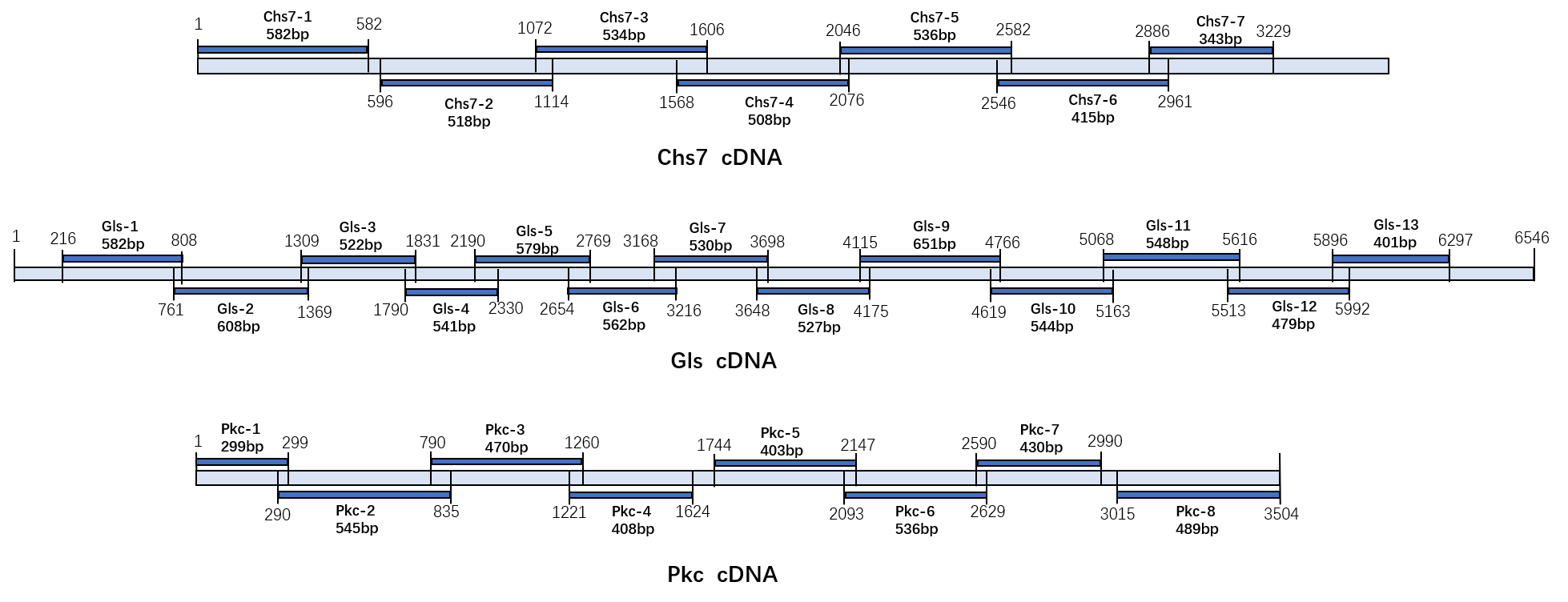

Supplement: Supplementary file 2 [file Image_1.TIF]

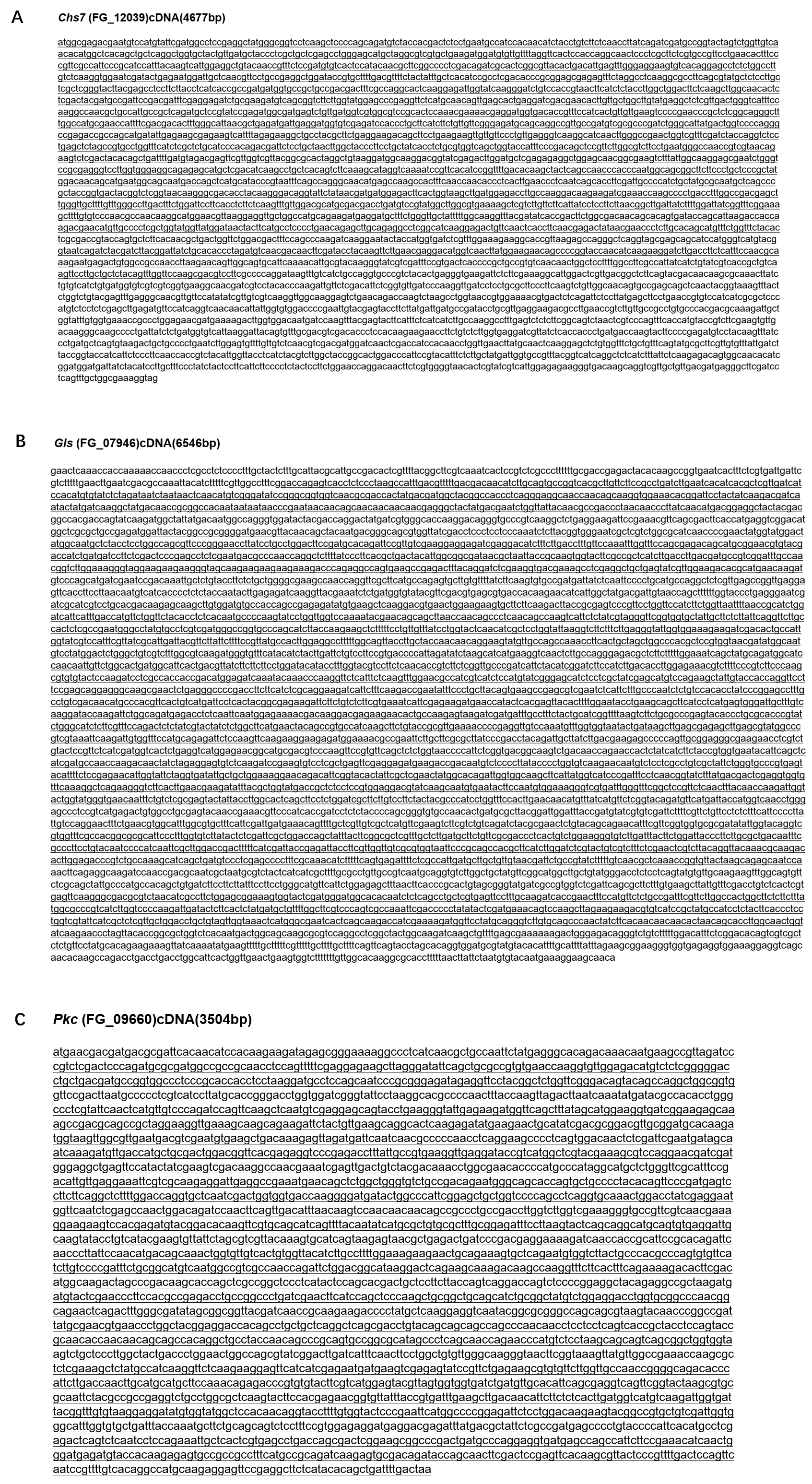

Supplement: Supplementary file 3 [file Image_2.TIF]

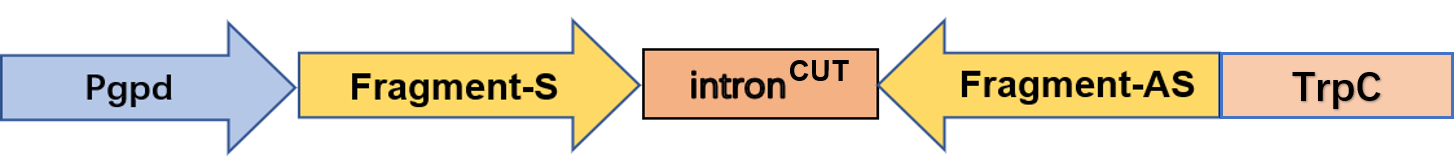

Supplement: Supplementary file 4 [file Image_3.TIF]
